# Administrative data ICD-10 diagnostic codes identifies most lab-confirmed SARS-CoV-2 admissions but misses many discharged from the Emergency Department

**DOI:** 10.1038/s41598-023-49501-7

**Published:** 2024-03-12

**Authors:** Cristiano S. Moura, Laurie J. Morrison, Corinne M. Hohl, Lars Grant, Louise Pilote, Autumn Neville, Jeffrey P. Hau, Sasha Bernatsky, and the Canadian COVID-19 Emergency Department Rapid Response Network (CCEDRRN) investigators, for the Network of Canadian Emergency Researchers, the Canadian Critical Care Trials Group

**Affiliations:** 1 McGill University, Montreal, QC Canada; 2 Department of Emergency Services, Sunnybrook Health Sciences Centre, Toronto, ON Canada; 3 Division of Emergency Medicine, Department of Medicine, University of Toronto, Toronto, ON Canada; 4 Vancouver General Hospital, Vancouver, BC Canada; 5 Research Institute of the McGill University Health Centre, Montreal, QC Canada; 6 University of British Columbia, Vancouver, BC Canada

**Keywords:** Health care, Epidemiology

## Abstract

We estimated the operating characteristics of ICD-10 code U07.1, introduced by the World Health Organization in 2020, to identify lab-confirmed SARS-CoV-2. CCEDRRN is a national research registry of adults (March 2020–August 2021) with suspected/confirmed SARS-CoV-2 identified in Canadian emergency departments (EDs) using chart review (symptoms, clinical information, and lab test results including SARS-CoV-2 polymerase chain reaction, PCR results). CCEDRRN data were linked to administrative hospitalization discharge and ED ICD-10 diagnostic codes (accessed centrally via the Canadian Institute for Health Information). We identified ICD-10 diagnostic codes in CCEDRRN participants. We defined lab-confirmed SARS-CoV-2 based on at least one positive PCR in the 0–14 days before the ED presentation and/or during hospitalization (in those admitted from ED). We performed separate analyses for CCEDRRN participants discharged from ED and those hospitalized from the ED. Additional analyses were stratified by province, sex, age, and (for hospitalized patients) timing of the first PCR test. The sensitivity of ICD-10 code U07.1 for a positive SARS-CoV-2 test was 93.6% (95% CI 93.0–94.1%) in those hospitalized from ED and 83.0% (95% CI 82.1–83.9%) in those discharged from the ED. Sensitivity was similar across provinces and demographics, but in each stratified analysis, values were higher in those hospitalized versus those discharged from ED. The ICD-10 diagnostic code for U07.1 within administrative data identified most lab-confirmed SARS-CoV-2 within persons hospitalized from ED, although a significant number of cases discharged from ED were missed. This should be considered when using administrative data for research and public health planning.

## Introduction

In March 2020, the World Health Organization (WHO) released a new International Classification of Diseases Revision 10 (ICD-10) code U07.1 (lab-confirmed SARS-CoV-2) to standardize the identification of cases.

One potential use of this new ICD coding would be to identify lab-confirmed SARS-CoV-2 for research and surveillance activities to support our understanding of the evolution of SARS-CoV-2 over time and across different jurisdictions. Our primary objective was to assess the operating characteristics of ICD-10 code U07.1 using the Canadian COVID-19 Emergency Department Rapid Response Network (CCEDRRN) registry linked to administrative diagnostic codes. CCEDRRN collected data very early in the pandemic when universal testing was not available to the community. Thus, the vast majority of people were only tested in hospital or the ED.

## Methods

### Study sample

CCEDRRN is a research registry of consecutive individuals with suspected/confirmed SARS-CoV-2 infection presenting to 51 urban and rural emergency departments (EDs) in eight Canadian provinces (British Columbia, Alberta, Manitoba, Saskatchewan, Quebec, Ontario, Nova Scotia, New Brunswick) from March 1, 2020–August 2021^1–3^. The registry obtained ethics approval to enroll participants into the registry with a waiver for informed consent, allowing us to capture a complete sample. Participants with suspected or confirmed COVID-19 presenting to one of the participating EDs were enrolled in the study using pre-defined clinical criteria (more details published elsewhere)^1–3^. In summary, patients were included in the study in two distinct periods (depending on the province and based on the availability of COVID-19 testing—see Supplemental Material, T﻿able A). The first period’s (covering the early phase of the pandemic up to April–May 2020) criteria included fever and one respiratory symptom (including flu-like illness, shortness of breath or cough) or presenting to the ED and tested for SARS-CoV-2 in the ED. The second period started on the date each province expanded testing criteria, allowing clinicians to test patients based on clinical suspicion or policy. Inclusion criteria in this period encompassed: (1) patients tested for SARS-CoV-2 in the ED or within 24 h of arrival and (2) patients presenting to the ED within 14 days of a positive SARS-CoV-2 test and presenting with clinical symptoms of COVID-19. In this period, elective, non-ED admissions were excluded. We excluded patients without available PCR tests for this study.

Standardized data abstracted from medical records includes demographics, symptoms, SARS-CoV-2 risk factors (e.g., travel, work, contacts), selected comorbidities, procedures, medications, SARS-CoV-2 RNA reverse transcription-polymerase chain reaction (PCR) testing, other lab results, and hospitalization details for those whose ED presentation resulted in admission. Of all ED visits in the CCEDRRN dataset, 95% have at least one PCR test available (an inclusion criteria for our current analyses), including negative, positive, and indeterminate/unknown results.

CCEDRRN has REB approval to link registry data (via each person’s unique provincial health number) with electronic administrative health databases with ICD-10 diagnostic codes (including U07.1) assigned during ED visits and during hospitalizations if admitted from the ED (here discharge data included deaths within the hospital stay). Administrative data were accessed via the Canadian Institute for Health Information, CIHI^4^. CIHI is an agency created by Canada’s federal, territorial, and provincial governments (except Quebec, which contributes limited data and thus is not included in our analyses—except for the overall description of the CCEDRRN registry)^4^. CIHI’s health system databases include the Discharge Abstract Database (DAD), which captures administrative health information about hospitalizations, and the National Ambulatory Care Reporting System (NACRS), capturing emergency and ambulatory care visits. In the period of our study, facilities from the province of British Columbia did not provide NACRS ICD code data; therefore, this province was not included in the analyses of individuals discharged from the ED.

The sensitivity, specificity, positive predictive value (PPV), and negative predictive value (NPV), along with 95% confidence intervals (CI), were estimated for the CCEDRRN-CIHI sample. We performed separate analyses for ED visits that resulted in discharge and those resulting in hospitalization from the ED.

### ICD-10 code U07.1

In the CCEDRRN-CIHI sample, we identified all administrative data U07.1 diagnostic codes from ED visits (and hospitalizations from ED when this occurred). We then assessed the performance of ICD-10 code U07.1 (laboratory-confirmed SARS-CoV-2) compared to our reference standard, PCR test results during our study time interval, i.e., 0–14 days before (or during) the ED visit or during hospital stay for those admitted from the ED. We limited our analyses to ED visits with at least one PCR test within that interval (except for the first few weeks of the pandemic, CCEDRRN enrollment required a PCR test; thus, about 95% of all CCEDRRN patients have at least one PCR SARS-CoV-2 test).

To analyze the operating characteristics of ICD-10 code U07.1 related to administrative data ED visit and/or hospital discharge diagnostic codes, we defined true positives (TP) as CCEDRRN-CIHI ED visits whose electronic administrative health data included ICD-10 diagnostic code U07.1 and had at least one positive PCR test at any time from 0 to 14 days before (or during) the ED visit or during the hospital stay in those admitted from the ED. Individuals with multiple tests within the period were considered a true case if at least one positive test. False positives (FP) were those ED visits that had an administrative data ICD-10 diagnostic code U07.1 and no positive PCR SARS-CoV-2 test result (but at least one test with negative or indeterminate/unknown result) documented in CCEDRRN that related to the 0–14 days before (or during) the ED visit and/or during hospitalization, in those admitted from ED. False negatives (FN) were CCEDRRN-CIHI ED visits without an administrative data ICD-10 diagnostic code U07.1 with at least one positive PCR test documented in CCEDRRN within the same interval. True negatives (TN) were those ED visits without ICD-10 code U07.1 and no positive PCR test (at least one test was done and recorded as negative or indeterminant).

### Stratified and sensitivity analyses

As noted earlier, in our main analyses, we performed separate analyses for CCEDRRN participants who were discharged from ED and those hospitalized from the ED. Additional stratified analyses were carried out to investigate potential differences in operating characteristics across provinces, across sex and age groups (< 50 years, 50–75 years, and > 75 years old), calendar periods, and selected comorbidities (asthma, pulmonary fibrosis, and chronic lung disease). For hospitalized patients, we also stratified by timing of the first PCR test.

### Ethics approval and consent to participate

The McGill University Research Ethics Board approved this study. The research ethics boards of participating institutions (see Supplement) reviewed and approved the study protocol with a waiver of informed consent for patient enrollment. All research was performed in accordance with relevant guidelines and regulations.

## Results

### CCEDRRN characteristics

The original CCEDRRN registry comprised 138,676 ED visits involving 112,995 participants enrolled between Mar. 1, 2020, and Aug. 27, 2021. Across all ED visits, the participant distribution was nearly equal between males and females, with a median age of 58 and an interquartile range of 39–74. Notably, three-quarters of participants came from the most populous provinces, Quebec, British Columbia and Ontario. Further details regarding these ED visits are provided in Table 1.

**Table 1:** Selected characteristics of 138,676 CCEDRRN ED visits, March 2020-August 2021.

Characteristic	N (%) or median (interquartile range, IQR)
Female sex, N (%)	68,607 (49.5)
Age in years, (IQR)	58 (39–74)
Age group, (N (%)	
Less 50 years	52,772 (38.1)
Between 50 and 75 years	52,165 (37.6)
75 years or more	33,739 (24.3)
Province, N (%)	
Alberta	31,227 (22.5)
British Columbia	34,225 (24.7)
New Brunswick	280 (0.20)
Nova Scotia	1201 (0.87)
Ontario	31,312 (22.6)
Quebec	35,372 (25.5)
Saskatchewan	5059 (3.7)
Calendar period of presentation, N (%)	
Before June 2020	18,451 (13.3)
June–Aug 2020	29,470 (21.3)
Sep–Dec 2020	34,079 (24.6)
Jan–Mar 2021	31,030 (22.4)
Apr–Aug 2021	25,646 (18.5)
Symptoms, N (%; patients may have more than one)	
Fever	30,986 (22.3)
Anosmia	2,471 (1.8)
Cough	42,173 (30.4)
Shortness of breath (dyspnea)	50,595 (36.5)
Others	61,630 (44.4)

The registry captured data on 161,591 PCR SARS-CoV-2 tests. Out of these tests, 128,032 (or 79.2%) yielded negative results, while 32,339 (20.0%) indicated a positive result. Additionally, 1220 tests (0.75%) returned an indeterminate/unknown result.

**Figure 1:**
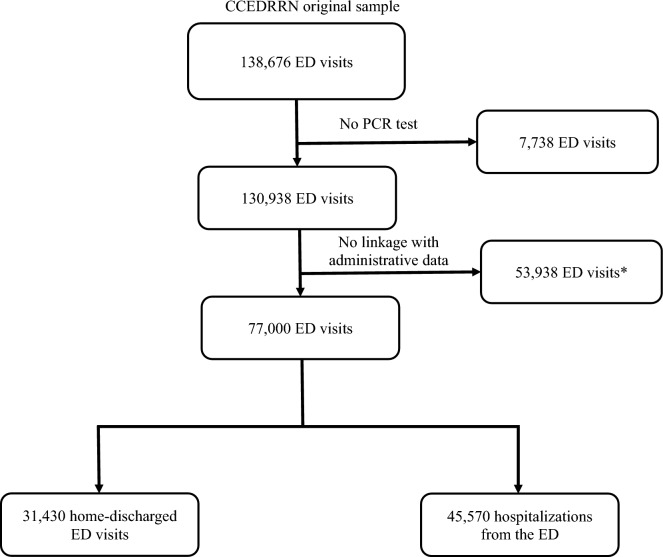
Flowchart of records selection. *Participants from British Columbia and Quebec could not be linked with administrative data as they do not provide the relevant data to the Canadian Institute for Health Information. Also, participants from British Columbia were included only in the hospitalization-based analysis, as this province does not provide data on ICD-10 codes for ED visits.

We studied 77,000 ED visits from the original CCEDRRN registry that had at least one PCR test (done during outpatient clinics, ED visits, or hospitalization) and linked to electronic administrative health data. The linkage included 31,430 home-discharged ED visits and 45,570 hospitalizations from the ED (see Fig. 1).

Among participants who were admitted to hospital from the ED, the sensitivity of diagnostic code U07.1 (from the linked administrative data) to detect lab-confirmed SARS-CoV-2 was 93.6% (95% CI 93.0–94.1%). The sensitivity of code U07.1 for lab-confirmed SARS-CoV-2 in CCEDRRN participants discharged from ED was 83.0% (95% CI 82.1–83.9%). The remaining operating characteristics for the main analyses are detailed in Table 2. Specificity, PPV, and NPV estimates were always better in individuals admitted from the ED. For example, in those hospitalized from the ED, the PPV of administrative data ICD diagnostic code U07.1 was 98.6% (95% CI 98.4–98.9%) versus 90.1% (95% CI 89.4–90.8%) for patients discharged from the ED. The sensitivity for those discharged from the ED was particularly low in the over-75 age group.

**Table 2:** Operating characteristics of ICD-10 code U07.1 (lab confirmed-SARS-CoV-2 infection) from emergency department (ED) and/or hospital diagnostic codes, with PCR testing as the reference standard.

Parameter	Hospitalized patients (95% CI)	ED patients (95% CI)
Sensitivity	93.6% (93.0–94.1%)	83.0% (82.1–83.9%)
Specificity	99.8% (99.7–99.8%)	97.5% (97.3–97.7%)
Positive predictive value	98.6% (98.4–98.9%)	90.1% (89.4–90.8%)
Negative predictive value	98.8% (98.7–98.9%)	95.5% (95.2–95.7%)

For hospitalized patients, results represent British Columbia (BC), Nova Scotia (NS), and Saskatchewan. For patients discharged from ED, necessary data on diagnostic codes was unavailable for BC, thus analyses represent NS and Saskatchewan.

Tables 3 and 4 present stratified analyses; sensitivity was similar across provinces and demographics, but in each stratified analysis, values were higher in hospitalizations versus those discharged from the ED. In the hospitalized sample, sensitivity was highest if the first PCR test occurred 0–14 days before ED presentation.

**Table 3:** Operating characteristics of ICD-10 code U07.1 (lab confirmed- SARS-CoV-2 infection) in administrative data, with PCR testing as the reference standard, stratified by province.

Parameter	Hospitalized patients (95% CI)	ED-discharged patients (95% CI)
Alberta		
Sensitivity	92.5% (91.5–93.6%)	84.5% (83.3–85.6%)
Specificity	99.8% (99.7–99.9%)	97.5% (97.2–97.8%)
Positive predictive value	99.1% (98.7–99.5%)	93.0% (92.1–93.8%)
Negative predictive value	97.9% (97.6–98.2%)	94.2% (93.7–94.6%)
Ontario		
Sensitivity	90.4% (88.8–92.1%)	91.4% (90.2–92.6%)
Specificity	99.8% (99.7–99.9%)	97.7% (97.4–98.0%)
Positive predictive value	97.2% (96.3–98.2%)	87.6% (86.3–89.0%)
Negative predictive value	99.2% (99.1–99.3%)	98.5% (98.2–98.7%)
Other provinces1		
Sensitivity	95.3% (94.6–96.0%)	54.9% (51.5–58.3%)
Specificity	99.7% (99.7–99.8%)	96.7% (96.0–97.3%)
Positive predictive value	98.8% (98.5–99.2%)	81.7% (78.5–84.9%)
Negative predictive value	98.9% (98.8–99.1%)	88.8% (87.7–89.9%)

^1^For hospitalized patients, represents British Columbia (BC), Nova Scotia (NS), and Saskatchewan. For patients discharged from the ED, necessary information on ICD codes was not available for BC, thus analyses represent NS and Saskatchewan.

**Table 4:** Operating characteristics of ICD-10 code U07.1 (lab confirmed- SARS-CoV-2 infection) in administrative data, with PCR testing as the reference standard, stratified by sex and age.

Parameter	Hospitalized patients (95% CI)	ED-discharged patients (95% CI)
Female sex		
Sensitivity	93.4% (92.5–94.3%)	83.2% (82.0–84.5%)
Specificity	99.8% (99.7–99.8%)	97.5% (97.2–97.8%)
Positive predictive value	98.4% (98.0–98.9%)	89.5% (88.4–90.5%)
Negative predictive value	98.9% (98.8–99.1%)	95.8% (95.5–96.1%)
Male sex		
Sensitivity	93.7% (92.9–94.4%)	82.8% (81.5–84.1%)
Specificity	99.8% (99.7–99.8%)	97.5% (97.2–97.8%)
Positive predictive value	98.8% (98.4–99.1%)	90.8% (89.7–91.8%)
Negative predictive value	98.7% (98.6–98.9%)	95.1% (94.7–95.5%)
Age < 50 years		
Sensitivity	93.0% (91.7–94.2%)	83.9% (82.7–85.0%)
Specificity	99.8% (99.6–99.9%)	97.4% (97.1–97.7%)
Positive predictive value	98.6% (98.0–99.2%)	90.6% (89.7–91.6%)
Negative predictive value	98.7% (98.0–99.0%)	95.3% (94.9–95.7%)
50–75 years		
Sensitivity	94.4% (93.7–95.2%)	83.6% (82.1–85.1%)
Specificity	99.7% (99.6–99.8%)	97.7% (97.3–98.0%)
Positive predictive value	98.6% (98.2–99.0%)	90.7% (89.5–92.0%)
Negative predictive value	98.8% (98.7–99.0%)	95.6% (95.2–96.0%)
> 75 years		
Sensitivity	92.6% (91.4–93.7%)	75.1% (71.6–78.6%)
Specificity	99.8% (99.8–99.9%)	97.5% (97.0–98.0%)
Positive predictive value	98.8% (98.3–99.2%)	83.9% (80.8–87.1%)
Negative predictive value	98.9% (98.7–99.1%)	95.8% (95.1–96.4%)

## Discussion

We found high sensitivity, specificity and PPV for ICD-10- diagnostic code U07.1 when positive PCR testing was considered the reference standard. ICD-10 code U07.1 had higher sensitivity, specificity and PPV in those hospitalized from the ED versus those discharged from the ED. This may be as expected, given that hospitalized patients would be sicker (often having a higher viral load, thus more likely to have a positive PCR test) and have more opportunities to have repeat PCR tests^5^. Given our prior finding that the sensitivity of PCR testing is very high in the ED and does not drop during the first few days of admission^5^, the poorer performance of code U07.1 in patients discharged from the ED likely reflects differences in how ICD-10 codes are assigned in ED versus hospitalization data. Specifically, a physician ordering a PCR in the ED may not have the result of that test at the time when the individual is discharged from the ED. This would presumably increase the chances of emergency physicians charting other diagnoses in the medical records, resulting in other ICD-10 codes being entered into administrative data instead of U07.1. The only other Canadian study evaluating the reliability of ICD-10 code U07.1 in identifying SARS-CoV-2 infections, with PCR as the reference standard, found results very similar to ours^6^.

CCEDRRN collected data very early in the pandemic when universal testing was not available to the community. Most people who were tested were tested in the hospital or the ED.

Of all patients in the reference dataset, only 2.3% have a self-reported SARS-COV-2 positive from the community within the 14 days of the ED visit and of these, 12% were reconfirmed with a positive test in the ED; however, 90% were discharged with confirmed COVID-19 as the diagnosis.

Thus, the vast majority of people were only tested in hospital or the ED. There is minimal overlap between the reference and the validation data set.

Four studies from the United States, again using positive PCR tests as the gold standard to validate ICD-10 discharge diagnostic codes, found results similar to ours^7–11^. One study from the Mass Gen Brigham system (which includes Massachusetts General Hospital, Brigham and Women’s Hospital, and other allied hospitals across Massachusetts) found a much lower sensitivity than in other studies^12^. Their estimates varied considerably over the study period, with the highest estimate (from May 2020) being 60.9% (57.3–64.4%)^12^. They attributed the lower sensitivity to delays in assigning discharge diagnostic codes, changes to PCR testing criteria and other factors^12^.

ICD-10 code U07.1 had only moderate agreement with PCR test positivity in those discharged from the ED. This is a potential concern, as many SARS-CoV-2-infected patients are discharged from the ED, representing a significant disease burden in the community. Our sensitivity estimates tended to be particularly low in older individuals (75+) discharged from the ED. This group is vulnerable to unfavourable outcomes after SARS-CoV-2 infection, and missing this group in public health surveillance or population-based research may considerably affect estimates. Health policy decisions relating to pandemic preparation, including resource planning, may not be optimal if based exclusively on ICD-10 code U07.1 administrative data, at least for patients discharged from the ED. This knowledge is important if we want a complete understanding of what ED and community resources may be required to manage future infectious disease crises (potentially including influenza surges and/or new health threats).

## Conclusion

In conclusion, ICD-10 diagnostic codes for U07.1 within administrative health data identified most lab-confirmed SARS-CoV-2 infections in patients admitted to the hospital from the ED. Administrative health data diagnostic codes were less sensitive for identifying lab-confirmed SARS-CoV-2 in patients discharged from the ED. This limitation is important to acknowledge if ICD code U07.1 is used for SARS-CoV-2 case detection, for research and public health purposes.

### Supplementary Information

Supplementary Information.

Supplementary Tables.

## Data Availability

The CCEDRRN Network policy is outlined here: https://www.ccedrrn.com/knowledge-users.
