# Supplementary material for: Administrative data ICD-10 diagnostic codes identifies most lab-confirmed SARS-CoV-2 admissions but misses many discharged from the Emergency Department
Source: Sci Rep. 2024 Mar 12;14:6008. doi: 10.1038/s41598-023-49501-7 (PMC10933440; doi:10.1038/s41598-023-49501-7)
Supplement: Supplementary file 2 — Supplementary Tables. [file 41598_2023_49501_MOESM2_ESM.docx]

Supplement Table A – Distribution of true positive, false positive, false negative, and true negative cases in the main and stratified analyses.

| **Analysis type** | **True Positive**  **N** | **False Positive**  **N** | **False Negative**  **N** | **True Negative**  **N** |
| --- | --- | --- | --- | --- |
| Main Analysis |  |  |  |  |
| Hospital | 6,598 | 91 | 454 | 38,427 |
| ED | 5,594 | 615 | 1,144 | 24,077 |
| Province stratification |  |  |  |  |
| Alberta - Hospital | 2,061 | 18 | 166 | 7,880 |
| Alberta - ED | 3,230 | 244 | 594 | 9,577 |
| Ontario - Hospital | 1,154 | 33 | 122 | 15,287 |
| Ontario - ED | 1,913 | 270 | 180 | 11,561 |
| Other Provinces - Hospital | 3,383 | 40 | 166 | 15,260 |
| Other Provinces - ED | 451 | 101 | 370 | 2,939 |
| Sex stratification |  |  |  |  |
| Male - Hospital | 3,888 | 48 | 262 | 20,679 |
| Male - ED | 2,667 | 271 | 555 | 10,682 |
| Female - Hospital | 2,710 | 43 | 192 | 17,743 |
| Female - ED | 2,927 | 344 | 589 | 13,393 |
| Age stratification |  |  |  |  |
| < 50y - Hospital | 1,512 | 22 | 114 | 8,882 |
| < 50y - ED | 3,245 | 336 | 624 | 12,657 |
| 50-75 years - Hospital | 3,189 | 45 | 188 | 15,811 |
| 50-75 years - Ed | 1,911 | 195 | 375 | 8,151 |
| >75 years - Hospital | 1,897 | 24 | 152 | 13,734 |
| >75 years - ED | 438 | 84 | 145 | 3,269 |
